# EIF5A2 controls ovarian tumor growth and metastasis by promoting epithelial to mesenchymal transition via the TGFβ pathway

**DOI:** 10.1186/s13578-021-00578-5

**Published:** 2021-04-07

**Authors:** Guannan Zhao, Wenjing Zhang, Peixin Dong, Hidemichi Watari, Yuqi Guo, Lawrence M. Pfeffer, Gabor Tigyi, Junming Yue

**Affiliations:** 1 Department of Pathology and Laboratory Medicine, College of Medicine, The University of Tennessee Health Science Center, Memphis, TN 38163 USA; 2 Center for Cancer Research, College of Medicine, The University of Tennessee Health Science Center, Memphis, TN 38163 USA; 3 Department of Genetics, Genomics & Informatics, College of Medicine, The University of Tennessee Health Science Center, Memphis, TN 38163 USA; 4 Department of Obstetrics and Gynecology, Hokkaido University Graduate School of Medicine, Sapporo, 060-8638 Japan; 5 People′s Hospital of Zhengzhou University, Zhengzhou, Henan China; 6 Department of Physiology, College of Medicine, The University of Tennessee Health Science Center, Memphis, TN 38163 USA

**Keywords:** EIF5A2, CRISPR, Cas9 nickase, Lentiviral vector, Ovarian cancer, Epithelial to mesenchymal transition, Orthotopic ovarian cancer mouse model

## Abstract

**Background:**

Epithelial to mesenchymal transition (EMT) contributes to tumor metastasis and chemoresistance. Eukaryotic initiation factor 5A2 (EIF5A2) is highly expressed in a variety of human cancers but rarely expressed in normal tissues. While EIF5A2 has oncogenic activity in several cancers and contributes to tumor metastasis, its role in ovarian cancer is unknown. In this study, we investigate whether EIF5A2 contributes to ovarian tumor metastasis by promoting EMT.

**Methods:**

To investigate the role of EIF5A2, we knocked out (KO) EIF5A2 using lentiviral CRISPR/Cas9 nickase in high invasive SKOV3 and OVCAR8 cells and overexpressed EIF5A2 in low invasive OVCAR3 cells using lentiviral vector. Cell proliferation, migration and invasion was examined in vitro ovarian cancer cells and tumor metastasis was evaluated in vivo using orthotopic ovarian cancer mouse models.

**Results:**

Here we report that EIF5A2 is highly expressed in ovarian cancers and associated with patient poor survival. Lentiviral CRISPR/Cas9 nickase vector mediated knockout (KO) of EIF5A2 inhibits epithelial to mesenchymal transition (EMT) in SKOV3 and OVCAR8 ovarian cancer cells that express high levels of EIF5A2. In contrast, overexpression of EIF5A2 promotes EMT in OVCAR3 epithelial adenocarcinoma cells that express relatively low EIF5A2 levels. KO of EIF5A2 in SKOV3 and OVCAR8 cells inhibits ovarian cancer cell migration and invasion, while its overexpression promotes cell migration and invasion in OVCAR3 adenocarcinoma cells. We further demonstrate that EIF5A2 promotes EMT by activating the TGFβ pathway and KO of EIF5A2 inhibits ovarian tumor growth and metastasis in orthotopic ovarian cancer mouse models.

**Conclusion:**

Our results indicate that EIF5A2 is an important controller of ovarian tumor growth and metastasis by promoting EMT and activating the TGFβ pathway.

**Supplementary Information:**

The online version contains supplementary material available at 10.1186/s13578-021-00578-5.

## Background

Ovarian cancer (OC) has the highest mortality rate among gynecological malignancies [1]. Early stage OC patients have no obvious symptoms and are often diagnosed only at later stages III and IV, when tumors have already metastasized to the peritoneal cavity or other abdominal organs. Early stage OC patients respond to chemotherapy, but eventually become resistant to chemotherapy. Although multi-modality treatment approaches applied in OC therapy include debulking surgery, chemotherapy, targeted therapy, and immunotherapy, the five-year survival rate remains poor at 35 to 40% [2–5]. The molecular mechanisms driving OC metastasis and chemoresistance remain unclear. Thus, it is of great importance to identify new predictive biomarkers for early diagnosis and develop new drugs to improve OC therapy.

Eukaryotic initiation factor 5A (EIF5A) is a eukaryotic translation initiation factor that participates in the initiation and elongation process in protein synthesis. EIF5A is the only known protein that undergoes hypusination through posttranslational modification. Deoxyhypusine synthase (DHPS) cleaves the polyamine spermidine and the 4-aminobutyl group is transferred to lysine residue 50 of EIF5A, which is subsequently hydroxylated by deoxyhypusine hydroxylase (DOHH) to facilitate EIF5A maturation [6–9]. There are two isoforms of EIF5A, EIF5A1 and EIF5A2, which share sequence similarity of 84% in mRNA and 94% protein [10]. EIF5A1 is expressed in the majority of cell types and required for embryonic development, while EIF5A2 is expressed only in specific cell types and is not required for embryonic development [11, 12]. Interestingly, EIF5A2 is aberrantly amplified or upregulated in various cancers including ovarian cancer, lung, pancreatic cancer, and hepatocellular carcinoma, and contributes to tumor growth and metastasis [7, 10, 13, 14]. Therefore, EIF5A2 is an attractive drug target for cancer therapy based on its aberrant expression in various cancer types. Although EIF5A2 is upregulated in ovarian cancer, its functional role has not been characterized at the mechanistic level. Previous studies demonstrated that EIF5A2 contributed to epithelial to mesenchymal transition (EMT) in colorectal, gastric, and breast cancer [15–17]. It is well known that EMT contributes to tumor initiation, progression, invasion, metastasis, EMT is regulated by multiple signaling pathways including ERK1/2, AKT, WNT in different cancers [18–20]. Although EMT contributes to tumor metastasis, the role of EMT in ovarian cancer is somewhat controversial due to the same expression levels of E-cadherin in the ovary and other distant metastatic organs [21]. However, accumulating evidence indicates that EMT plays an important role in ovarian tumor metastasis [22–26], Our previous studies showed that TGFβ promoted EMT in ovarian cancer cells [27], and BIRC5 (survivin) expression activates the TGFβ pathway and promotes EMT and ovarian tumor metastasis in orthotopic ovarian cancer mouse models [28].

In the present study we provide evidence that EIF5A2 contributes to ovarian tumor growth and metastasis by promoting EMT via activation of the TGFβ pathway.

## Materials and methods

### Cell culture

Ovarian cancer cell line SKOV3 was purchased from ATCC and cultured in Dulbecco’s Modified Eagle Medium (DMEM) supplemented with 10% FBS (Hyclone; Logan, UT), 100 U/ml penicillin/streptomycin (Invitrogen; Carlsbad, CA). OVCAR3 and OVCAR8 cell lines were purchased from National Cancer Institute and cultured in RPMI 1640 with 10% FBS (Hyclone; Logan, UT), 1% penicillin/streptomycin (Invitrogen; Carlsbad, CA). All cell lines were grown at 37 °C with 5% CO_2_. Cell lines were authenticated using Short Tandem Repeat (STR) analysis by ATCC and tested negative for mycoplasma using the luciferase assay (Lonza, Allendale, NJ).

### Lentiviral vector production

The lentiviral CRISPR/Cas9 nickase-mediated EIF5A2 gene editing vectors were constructed by annealing two gRNA oligonucleotide pairs and subcloning them into BsmII site of lentiviral vector Lentiguide-puro vector (#52,963, Addgene), and gRNAs were driven by human U6 promoter. Two gRNA sequences, 5′ AACGGCTTCGTGGTGCTCAA and 5′ CGCAAGGCCGAGCACTGCAT were designed to target exon 1 of EIF5A2 gene. TGFβR2 Knockdown (KD) CRISPR/Cas9 nickase vectors were constructed as described previously [28]. EIF5A2 lentiviral overexpression vector was purchased from Applied Biological Materials Inc. (Richmond, Canada). Lentivirus was produced by packaging in 293FT cells as we reported previously [29]. EIF5A2 KO and TGFβR2 KD stable cell lines were established by transducing SKOV3 and OVCAR8 ovarian cancer cells with the lentiviral CRISPR/Cas9 nickase vector and selected with 2 μg/ml puromycin and 10 μg/ml blasticidin. LentiCas9-blast was used as the control vector without gRNAs. The EIF5A2 stable expression and control cells were established by transducing OVCAR3 with lentiviral EIF5A2 and empty control vectors and selected with 2 μg/ml puromycin.

### MTT cell proliferation assay

SKOV3, OVCAR8 EIF5A2 KO or OVCAR3 expressing and corresponding control cells (3000/well) were plated into 96-well plates. Cell proliferation was measured at 24, 48 and 72 h using the MTT proliferation assay kit from ATCC (Manassas, VA) according to the instruction of the manufacturer.

### Clonogenic cell survival assay

6-well plates were seeded with 400 EIF5A2 KO SKOV3, EIF5A2 KO OVCAR8, EIF5A2 overexpressing (OE) OVCAR3 cells, and appropriate control cells. Plates were cultured for 2 weeks, fixed with 70% ethanol, and stained with crystal violet. Colonies were counted from three different wells and compared to corresponding controls.

### Cell migration assay

The cell migration assay was performed using the Transwell chambers from BD Falcon™ (San Jose, CA) as described previously [30]. Briefly, EIF5A2 KO SKOV3, EIF5A2 KO OVCAR8, EIF5A2 OE OVCAR3 cells, and corresponding control cells (5 × 10^4^) in 300 µl serum-free culture media were added into the upper chamber with 10% FBS in the lower chamber inserted in 24-well plates and cultured for 8 h. The migrated cells on the lower side of the membranes were fixed with methanol and then stained with crystal violet and counted.

### Cell invasion assay

Cell invasion assay was performed as described previously [30]. Briefly, EIF5A2 KO SKOV3, EIF5A2 KO OVCAR8, EIF5A2 OE OVCAR3, and the appropriate control cells (3 × 10^5^) were seeded in 300 µl serum-free culture medium onto transwell plates precoated with Matrigel (BD BioSciences, San Jose, CA). The invaded cells were stained for 10 min with hematoxylin and eosin (H&E) following methanol fixation for 20 min and at least three different fields were counted.

### SMAD dependent reporter gene assay

The EIF5A2 KO SKOV3, EIF5A2 KO OVCAR8 cells, EIF5A2 OE OVCAR3, and the corresponding control cells were transduced with the lentiviral vector pGF-SMAD2/3/4-mCMV-Luciferase-EF1a-puro (System Biosciences, CA) containing SMAD2/3/4 transcriptional response elements (TRE) and treated with 6 ng/ml TGFβ for 12 h. The luciferase activity was measured and normalized by comparing to control cells.

### Immunofluorescence staining

Formalin-fixed paraffin-embedded (FFPE) sections of de-identified ovarian serous carcinoma were obtained from the Tissue Services Core of the University of Tennessee Health Science Center (UTHSC). H&E staining was performed by Histology Core of UTHSC. Immunofluorescent staining was carried out as described previously [30]. The primary antibodies EIF5A2, Cytokeratin-7 (1:200) were purchased from Abcam and Vimentin (1:200 dilution) were purchased from Cell Signaling (Danvers, MA). Alexa 488- or 594- conjugated goat anti-rabbit or anti-mouse antibodies were purchased from Invitrogen (Carlsbad, CA). Cell nuclei were counterstained with DAPI (Vector Laboratories, Inc.; Burlingame, CA). Images were captured with the Nikon NIS Element software.

### Western blot

Western blot (WB) was performed as described previously [30]. Briefly, ovarian cancer cells were collected in RIPA buffer (Thermo Scientific; Rockford, IL) containing 1% Halt Proteinase Inhibitor Cocktail (Thermo Scientific; Rockford, IL). Equal amounts of protein (100 μg/lane) were loaded onto 10% SDS-PAGE gels and transferred onto nitrocellulose membranes. The membranes were blocked with 5% nonfat milk for 1 h and incubated with primary antibodies against EIF5A2, Cytokeratin-7(Abcam), GAPDH (Santa Cruz; St. Louis, MO), Vimentin, Ecadherin, β-catenin, snail2, SMAD2 or p-SMAD2 (Cell Signaling).

### Orthotopic ovarian cancer mouse model

To examine whether EIF5A2 contributes to primary ovarian tumor growth and metastasis, we injected intrabursally 5 × 10^5^ EIF5A2 KO and control SKOV3 cells transduced with lentiviral luciferase reporter vector and into 2 month–old immunocompromised NOD.Cg *Prkdcscid Il2rgtm1Wjl*/SzJ (NSG) female mice (n = 5/group). Ovarian tumor growth and metastasis was monitored using a Xenogen live animal imaging system once a week. Mice were sacrificed at 5 weeks after cell injection, and primary ovarian tumors and metastatic organs were harvested for histology and immunostaining.

## Results

### EIF5A2 expression is amplified and upregulated in ovarian adenocarcinomas and is predictive of patient poor survival

**Fig. 1:**
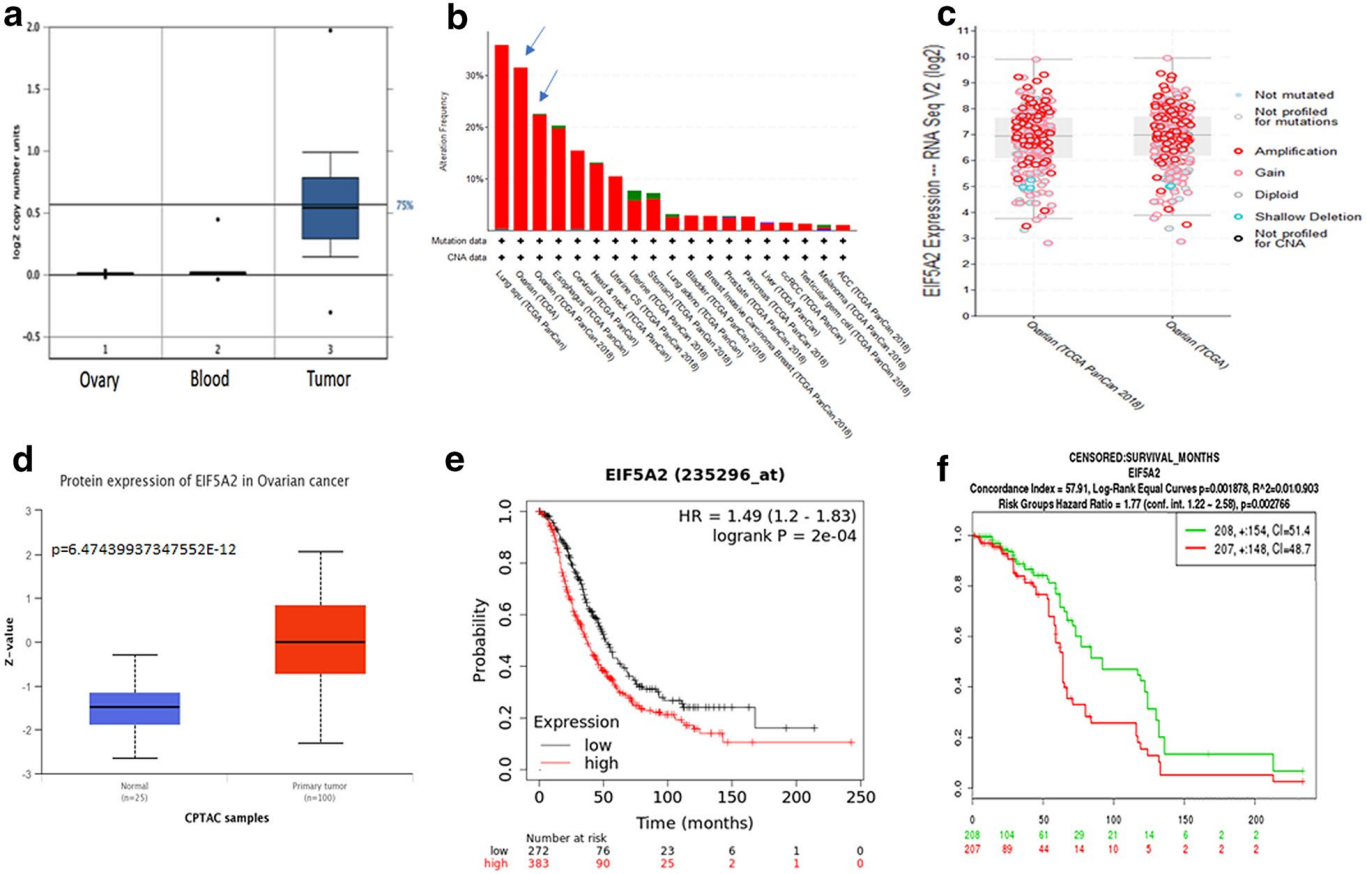
EIF5A2 expression is upregulated or amplified in ovarian cancer and associated with patient poor survival. **a** EIF5A2 copy numbers in normal and cancer tissues. 1: normal ovaries (N = 130); 2: normal blood (n = 431). 3: OC (n = 607). **b** EIF5A2 is amplified in majority of cancer types from in TCGA database. **c** EIF5A2 copy number is correlated with EIF5A2 mRNA expression from RNA-seq in two different datasets including TCGA PanCan and firehose legacy. **d** Protein expression of EIF5A2 in normal and ovarian cancer tissues. Normal ovaries (N = 25), Ovarian cancer (N = 100). **e** EIF5A2 expression is associated with overall survival of ovarian cancer patients in Kaplan Meier Plotter database **f** Ovarian cancer patients displayed significantly reduced patient survival in the high-risk compared to the low-risk group

To assess the expression of EIF5A2 in ovarian cancer, we analyzed 607 serous ovarian carcinomas and 561 normal tissues including 130 ovaries, plus 431 blood samples in the Oncomine database [31]. EIF5A2 copy numbers were significantly amplified in ovarian tumors as compared to normal tissues (*p* = 1.94E-197) (Fig. 1a). We also analyzed EIF5A2 expression in multiple cancer types from TCGA database, and EIF5A2 was amplified across multiple cancer types with the highest percentage found in specimens from lung and ovarian cancer patients (Fig. 1b). We further examined the correlation of EIF5A2 copy number and mRNA expression in two different datasets of TCGA database including 629 serous carcinomas from Firehose Legacy and 608 from PanCancer Atlas. The copy number alteration of EIF5A2 correlated with mRNA expression based on RNA-seq data (Fig. 1c). We also examined EIF5A2 expression from Clinical Proteomic Tumor Analysis Consortium (CPTAC) database including 100 specimens from ovarian cancer patients and 25 normal ovaries that also showed significantly higher protein expression in ovarian cancer tissues than that in normal ovaries (Fig. 1d). We then examined the expression of EIF5A2 in highly-invasive SKOV3 and OVCAR8 ovarian cancer cells, and low-invasive OVCAR3 cells using WB blot. EIF5A2 expression was significantly higher (p  <  0.001) in both SKOV3 and OVCAR8 cells than that in OVCAR3 cells (Additional file 1: Fig. S1A). To verify the expression of EIF5A2 in ovarian cancer tissues, we performed immunofluorescent staining on sections from three ovarian serous carcinoma patients that had been verified by H&E staining. EIF5A2 staining was strong in the cytoplasm of tumor cells but remained weak in the adjacent normal tissues (Additional file 1: Fig. S1B). To determine whether EIF5A2 expression is associated with patient overall survival (OS), we examined the correlation of EIF5A2 expression with ovarian cancer patient survival based on the Kaplan Meier Plotter database of 655 ovarian cancer samples including 383 with high EIF5A2 and 272 with low expression [32]. The OS was significantly reduced in patients with EIF5A2 high expression as compared to low expression patients (Fig. 1e). We also examined an additional 415 ovarian carcinoma including 207 EIF5A2 high and 208 low in the SurvExpress database [33]. EIF5A2 expression was analyzed based on risk groups. We found that EIF5A2 expression was significantly higher in the high-risk group than that in low-risk group (Additional file 1:Fig. S1c), whereas the OS was significantly reduced in high-risk compared to low-risk group (Fig. 1f). Thus, EIF5A2 expression correlated well with poor ovarian patient survival indicating a potential predictive biomarker for ovarian cancer diagnosis.

### EIF5A2 promotes EMT in ovarian cancer cells

**Fig. 2:**
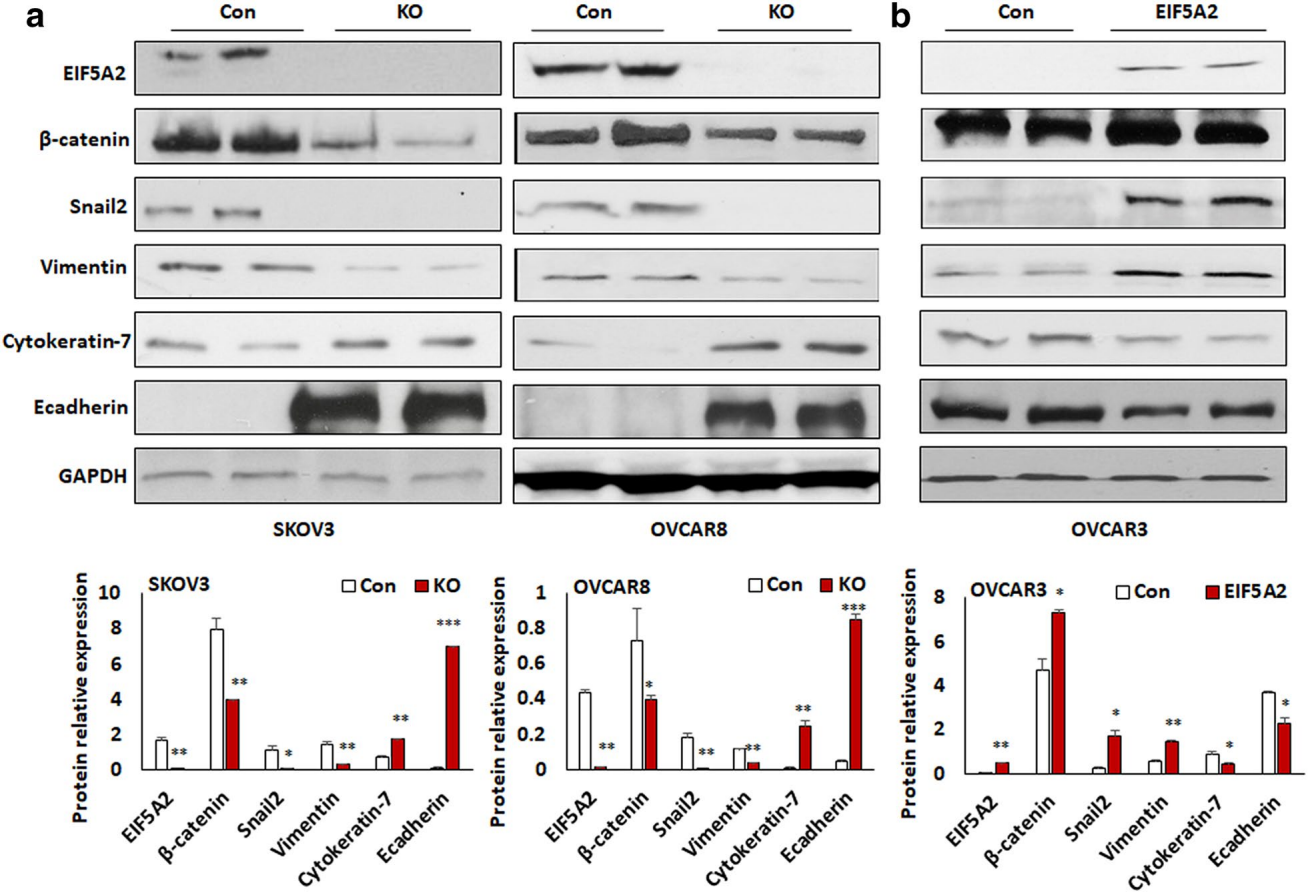
Disruption of EIF5A2 expression using lentiviral CRISPR/Cas9 nickase mediated editing resulted in the inhibition of EMT in ovarian cancer cells. **a** Western blot analysis of EIF5A2 and EMT markers in EIF5A2 KO and control (Con) SKOV3 and OVCAR8 cells. **b** Western blot analysis of EIF5A2 and EMT markers in EIF5A2 expression and control OVCAR3 cells. Band intensity was measured using Image J and statistically analyzed. One representative western blot was presented from three similar independent experiments. (*p < 0.05, **p < 0.01,*** p < 0.001)

Based on the expression level of EIF5A2 in these three lines, we set out to determine whether EIF5A2 contributes to EMT phenotypic switch by knocking out EIF5A2 in highly invasive SKOV3 and OVCAR8 cells using lentiviral CRISPR/Cas9 nickase vector, and conversely overexpressing it in low-invasive OVCAR3 cells using lentiviral vector under the control of EF1α promoter. EIF5A2 was undetectable in both SKOV3 and OVCAR8 KO cells, while EIF5A2 expression was substantially elevated in EIF5A2 OE OVCAR3 cells compared to control cells. The EMT markers including the epithelial cell marker cytokeratin-7 and E-cadherin were upregulated, whereas the mesenchymal markers β-catenin, vimentin, and snail2 were downregulated in both SKOV3 KO and OVCAR8 KO compared to control cells (Fig. 2a). In contrast, overexpression of EIF5A2 in OVCAR3 cells resulted in the downregulation of epithelial cell markers cytokeratin-7 and E-cadherin, whereas the mesenchymal markers β-catenin, vimentin, and snail2 were increased compared to control cells (Fig. 2b).

### Loss of EIF5A2 expression inhibits ovarian cancer cell proliferation and clonogenicity

**Fig. 3:**
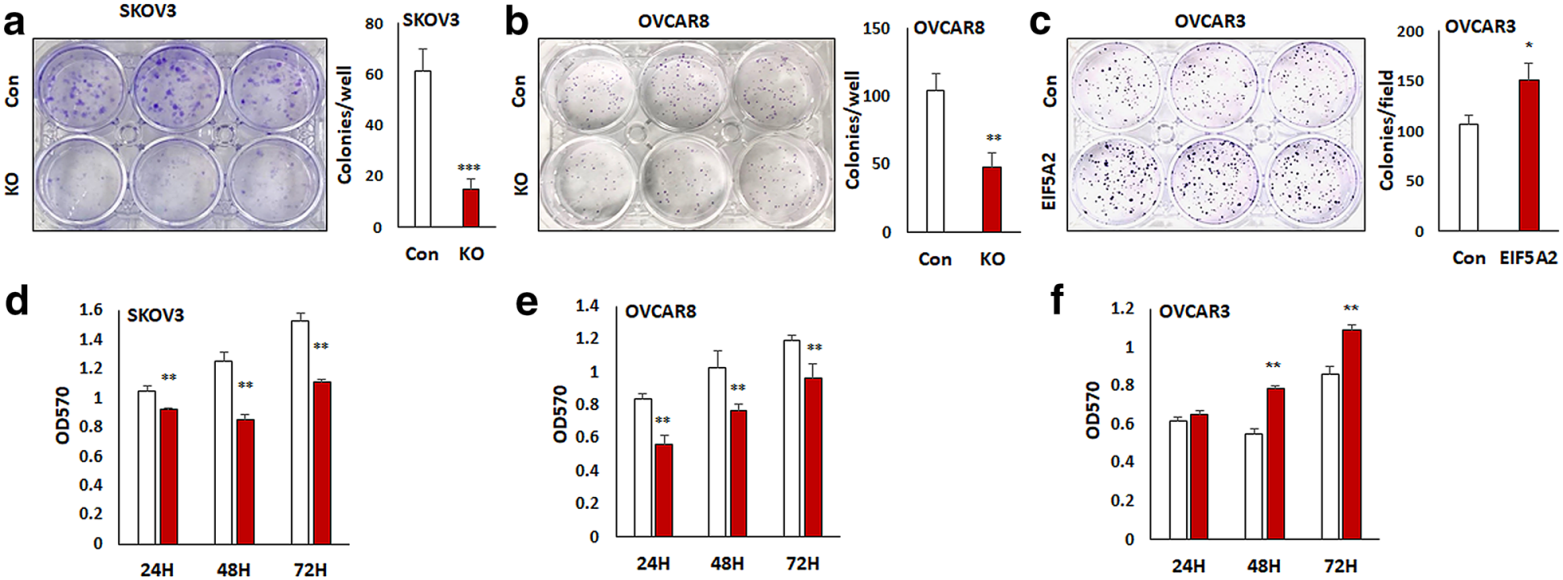
Disruption of EIF5A2 expression inhibition of cell proliferation and survival in ovarian cancer cells. **a**, **b** Cell colonies in EIF5A2 KO and control SKOV3 and OVCAR8 cells. **c** Cell colonies in EIF5A2 expression and control OVCAR3 cells. **d**, **e** Cell proliferation in EIF5A2 KO and control SKOV3 and OVCAR8 cells was determined by MTT assay. **f** Cell proliferation in EIF5A2 expressing and control OVCAR3 cells was determined by MTT assay (*P < 0.05; **P < 0.01***P < 0.001)

To determine the role of EIF5A2 in ovarian cancer cells, we examined cell survival in EIF5A2 KO SKOV3 and OVCAR8 cells, EIF5A2 OE OVCAR3 cells and control cells using cell colony formation assay. KO of EIF5A2 significantly reduced colony formation in both SKOV3 (Fig. 3a) and OVCAR8 cells (Fig. 3b), whereas OE of EIF5A2 in OVCAR3 cells significantly increased the number of colonies (Fig. 3c) compared to control cells. We also examined cell proliferation rate of EIF5A2 KO SKOV3, EIF5A2 KO OVCAR8 cells, and EIF5A2 OE OVCAR3 cells using the MTT assay. KO of EIF5A2 significantly reduced the rate of cell proliferation compared to controls at all three time points (24, 48, and 72 h) in both KO cell lines (Fig. 3d, e). In contrast, OE of EIF5A2 promoted cell proliferation at 48 and 72 h but no significant difference in the cell number relative to control was found at 24 h (Fig. 3f).

### Loss of EIF5A2 expression inhibits ovarian cancer cell migration and invasion

**Fig. 4:**
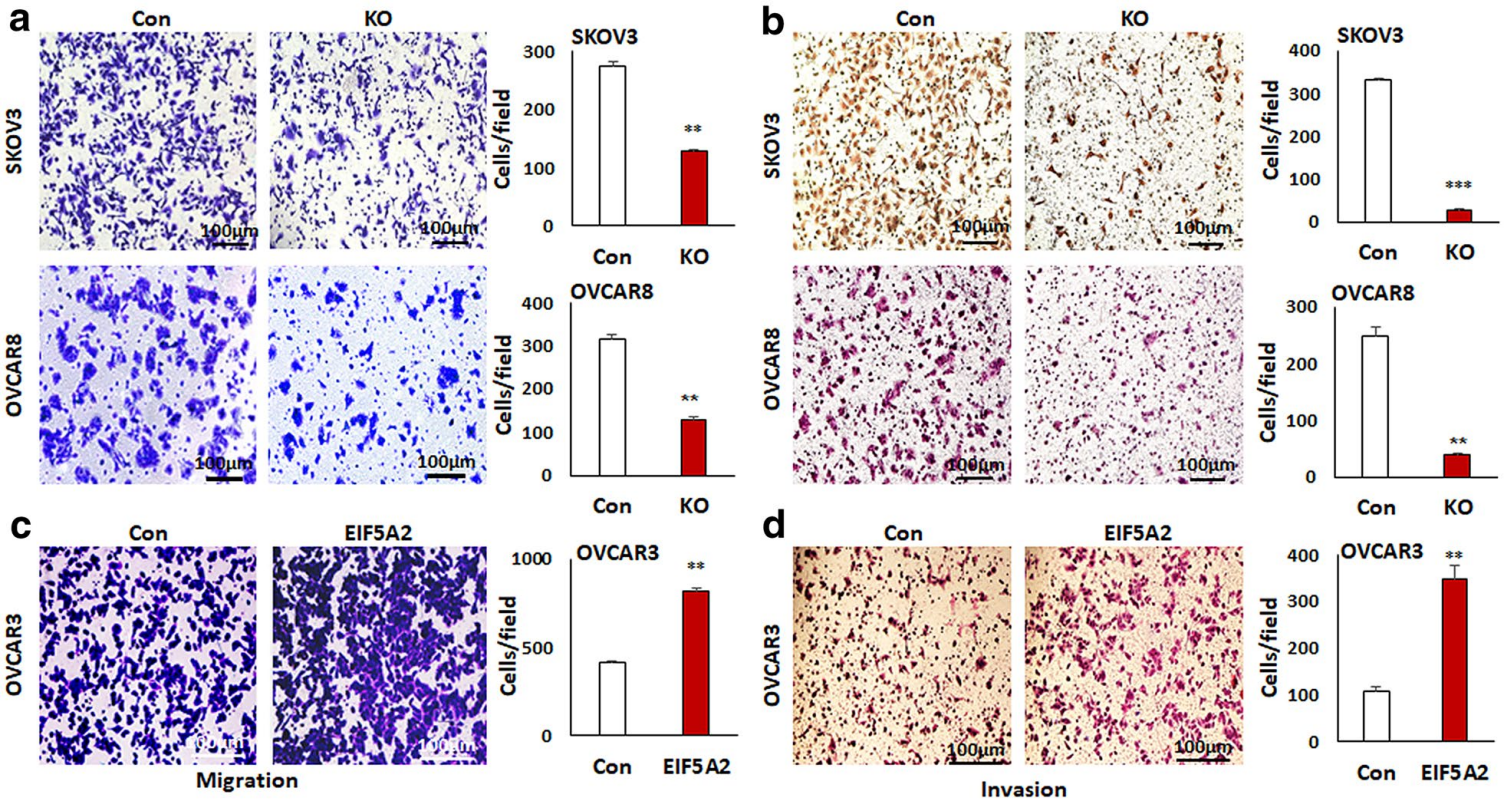
Disruption of EIF5A2 expression led to the inhibition of cell migration and invasion in ovarian cancer cells. **a** Cell migration in EIF5A2 KO and control SKOV3 or OVCAR8 cells was examined using the Transwell plates, and migrated cells were stained with crystal blue and counted from at least three different fields. **b** Cell invasion in both EIF5A2 KO and control SKOV3 or OVCAR8 cells was examined using Matrigel-coated plates, and invaded cells were stained with H&E. and counted from at least three different fields **c** Cell migration in EIF5A2-expressing and control OVCAR3 cells was examined using transwell plates, and migrated cells were stained with crystal blue and counted from at least three different fields. **d** Cell invasion in EIF5A2-expressing OVCAR3 and control was examined using Matrigel-coated plates, and invaded cells were stained with H&E. and counted from at least three different fields (**P < 0.01; ***P < 0.001)

EMT is an essential step in tumor cell migration and invasion. Since we found that EIF5A2 promoted EMT in ovarian cancer cells (Fig. 2), we also determine the role of EIF5A2 in migration and invasion of ovarian cancer cells using Transwell plates. KO of EIF5A2 significantly inhibited migration (Fig. 4a) and invasion (Fig. 4b) in SKOV3 and OVCAR8 cells, whereas OE of EIF5A2 significantly enhanced migration (Fig. 4c) and invasion in OVCAR3 (Fig. 4d).

### EIF5A2/TGFβ forms a positive feedback loop in promoting EMT in ovarian cancer cells

**Fig. 5:**
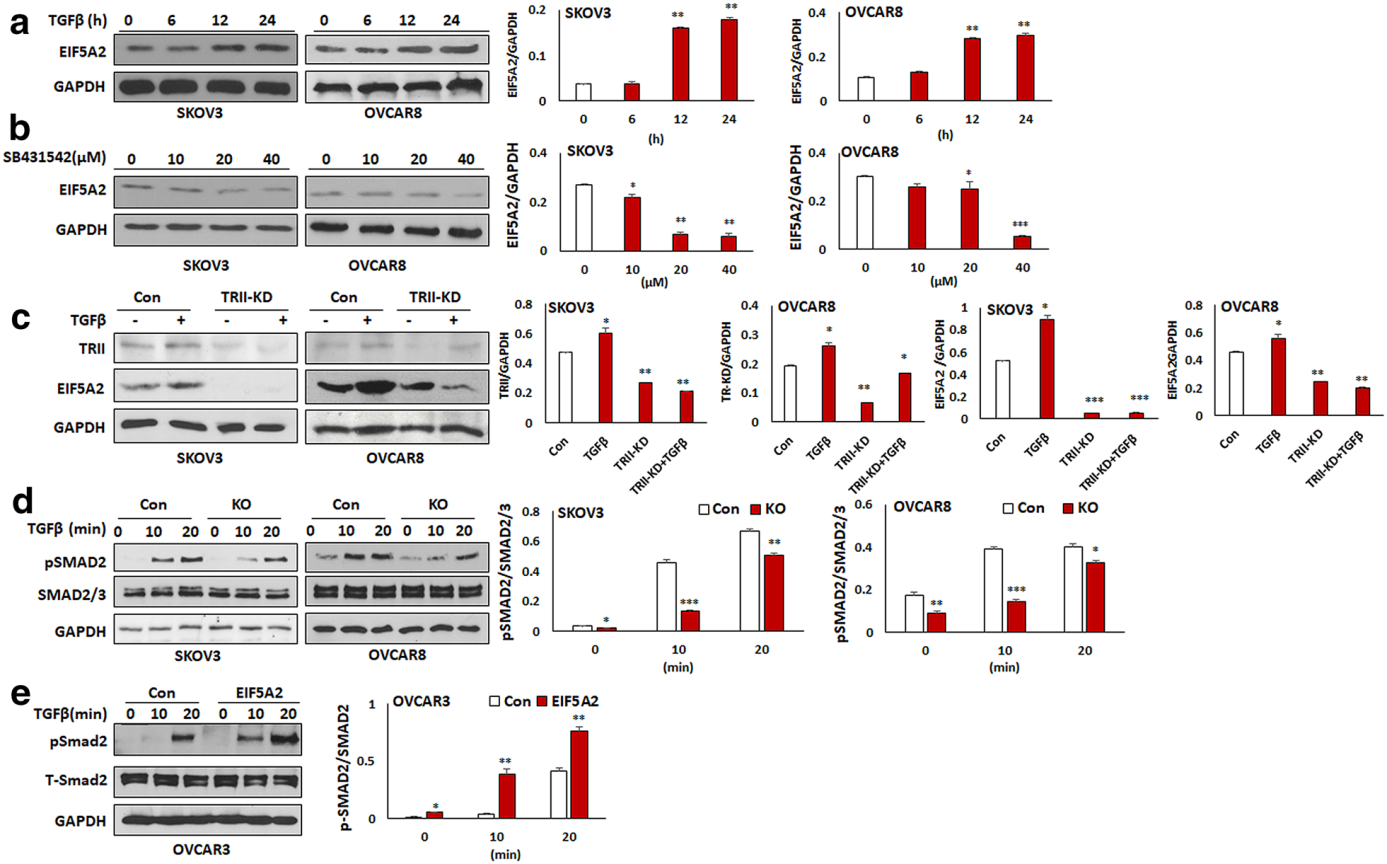
The association of EIF5A2 with the TGFβ pathway and inhibition of EIF5A2 attenuated TGFβ signaling pathway in ovarian cancer cells**. a** TGFβ induced EIF5A2 expression in SKOV3 and OVCA8 cells at the indicated time points as detected by Western blot. **b** TGFβR1/2 inhibitor SB431542 inhibited EIF5A2 expression in SKOV3 and OVCAR8 cells following 24 h treatment as detected by Western blot. **c** Western blot analysis of EIF5A2 and TGFβRII in TGFβR2 KD and control SKOV3 and OVCAR8 cells following 6 ng/ml TGFβ treatment for 24 h, respectively. **d**, **e** Western blot analysis of phospho- and total SMAD2 in EIF5A2 KO and control SKOV3 and OVCAR8 cells or in EIF5A2 expressing and control OVCAR3 following 6 ng/ml TGFβ treatment at the indicated time points. (*p < 0.05; **P < 0.01; ***P < 0.001)

We reported previously that TGFβ promoted EMT in ovarian cancer cells [27]. To understand how EIF5A2 contributed to EMT in ovarian cancer cells, we examined the potential co-regulation between EIF5A2 and TGFβ pathway in ovarian cancer cells by exposing both SKOV3 and OVCAR8 cells with 6 ng/ml TGFβ. As shown in Fig. 5a, TGFβ induced EIF5A2 expression in a time-dependent manner in both cell lines. We also treated both SKOV3 and OVCAR8 cells for 24 h with different doses of the TGFβR1/2 inhibitor SB431542 and found that EIF5A2 expression was inhibited in a dose-dependent manner in these two cell lines (Fig. 5b).

In addition to the pharmacological approach, we used a genetic approach by KD TGFβ receptor 2 (TGFβR2) using a lentiviral CRISPR/Cas9 nickase vector and then treated both types of cells with 6 ng/ml TGFβ for 24 h and determined EIF5A2 expression. KD of TGFβR2 significantly reduced EIF5A2 protein levels in both lines of cells. In contrast, TGFβ induced EIF5A2 expression in control, but not in the KD cells (Fig. 5c), indicating that TGFβ promoted EIF5A2 expression.

To examine how EIF5A2 regulates the TGFβ pathway, EIF5A2 KO SKOV3, EIF5A2 KO OVCAR8 cells, and control cells were treated with 6 ng/ml TGFβ. Phospho- and total SMAD2 in EIF5A2 KO and control ovarian cancer cells was examined by WB. Loss of EIF5A2 attenuated the TGFβ pathway as shown by the reduced level of phospho-SMAD2 in both SKOV3- and OVCAR8-KO cells compared to control cells (Fig. 5d). We further examined the TGFβ signaling by treating both EIF5A2 OE and control OVCAR3 cells with 6 ng/ml TGFβ. As expected, OE of EIF5A2 activated the TGFβ pathway as shown by increased phospho-SMAD2 in OE OVCAR3 cells compared to control cells (Fig. 5e).

**Fig. 6:**
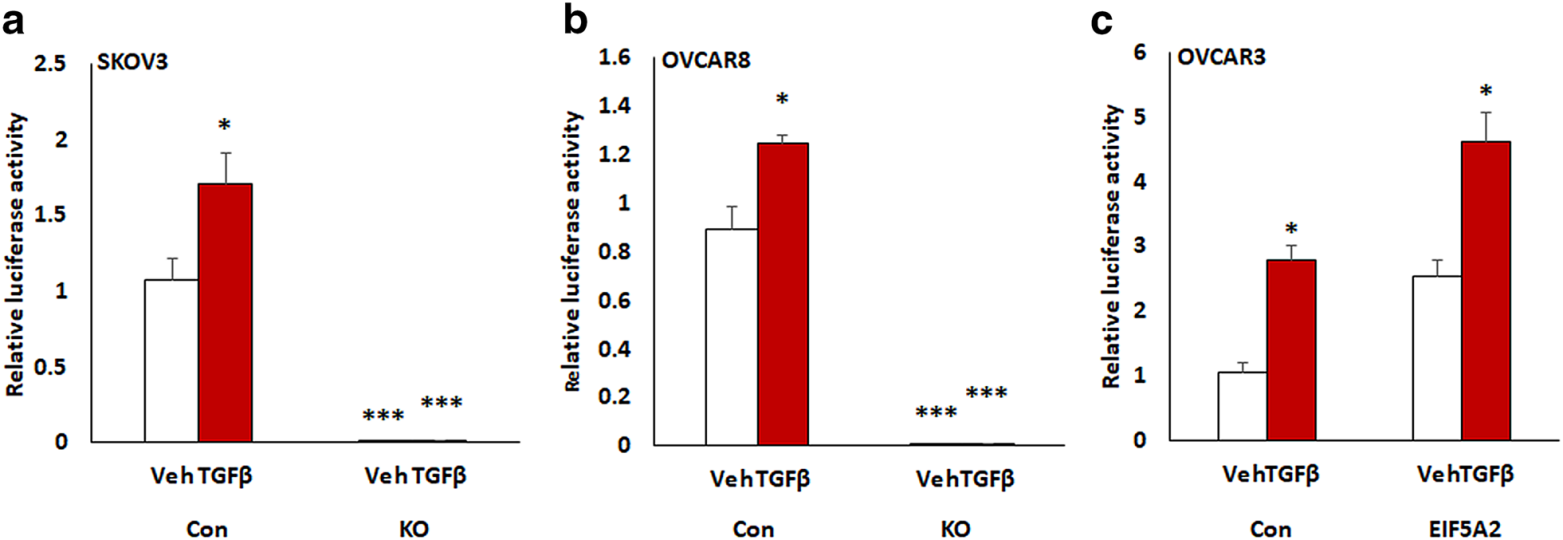
SMAD dependent reporter gene luciferase activity. Luciferase activity in EIF5A2 KO and control SKOV3 and OVCAR8 cells or EIF5A2 expressing and control OVCAR3 cells transduced with pGreenFire1-SMAD2/3/4-GF-EF1-puro lentiviral vector following 6 ng/ml TGFβ treatment for 12 h (*p < 0.05, *** p < 0.001)

To further examine the interaction of EIF5A2 and TGFβ signaling pathway, we transduced EIF5A2 KO, OE and control cells with a lentiviral luciferase reporter construct containing six SMAD2/3/4 response elements upstream of CMV mini-promoter and then treated transduced cells with 6 ng/ml TGFβ for 12 h. Loss of EIF5A2 significantly inhibited the luciferase activity in both SKOV3- and OVCAR8-KO cells while OE of EIF5A2 enhanced luciferase activity in OVCAR3 cells (Fig. 6). Our data indicate that EIF5A2 forms a positive feedback loop with TGFβ pathway in ovarian cancer cells.

### Loss of EIF5A2 suppressed primary ovarian tumor growth and metastasis by inhibiting EMT and attenuating the TGFβ pathway in an orthotopic ovarian mouse model

**Fig. 7:**
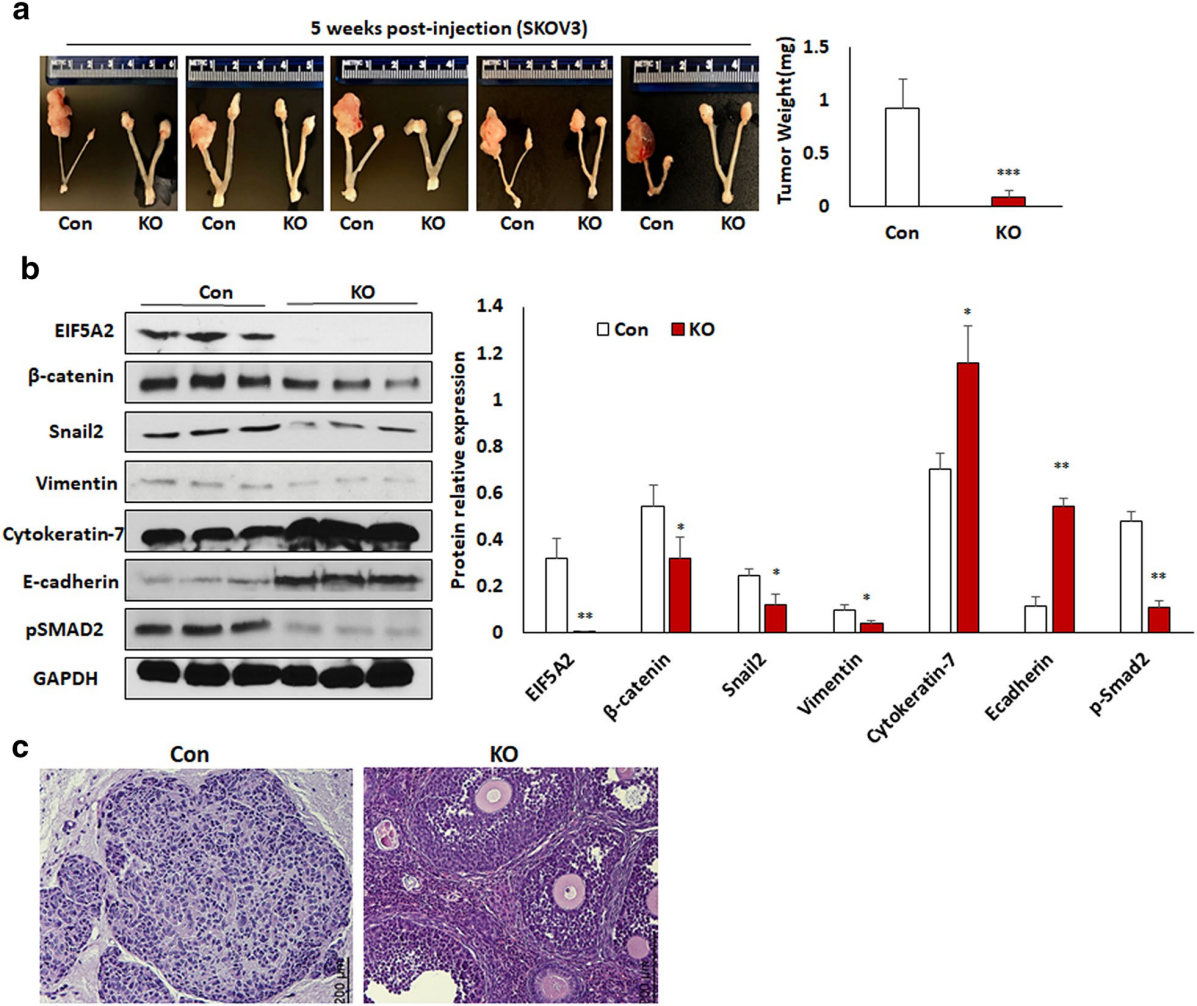
Knockout of EIF5A2 using lentiviral CRISPR/Cas9 nickase vector suppressed primary ovarian tumor growth in an orthotopic ovarian mouse model. **a** Primary ovarian tumors at one month following intrabursal injection of EIF5A2 KO and control SKOV3 cells (n = 5). **b** Western blot and densitometry analysis of EIF5A2, p-Smad2 and EMT markers from primary tumor of mice xenografted with EIF5A2 KO and control cells C. Sections of primary ovarian tumors were stained with H&E. (*p < 0.05, **p < 0.01, ***p < 0.001)

**Fig. 8:**
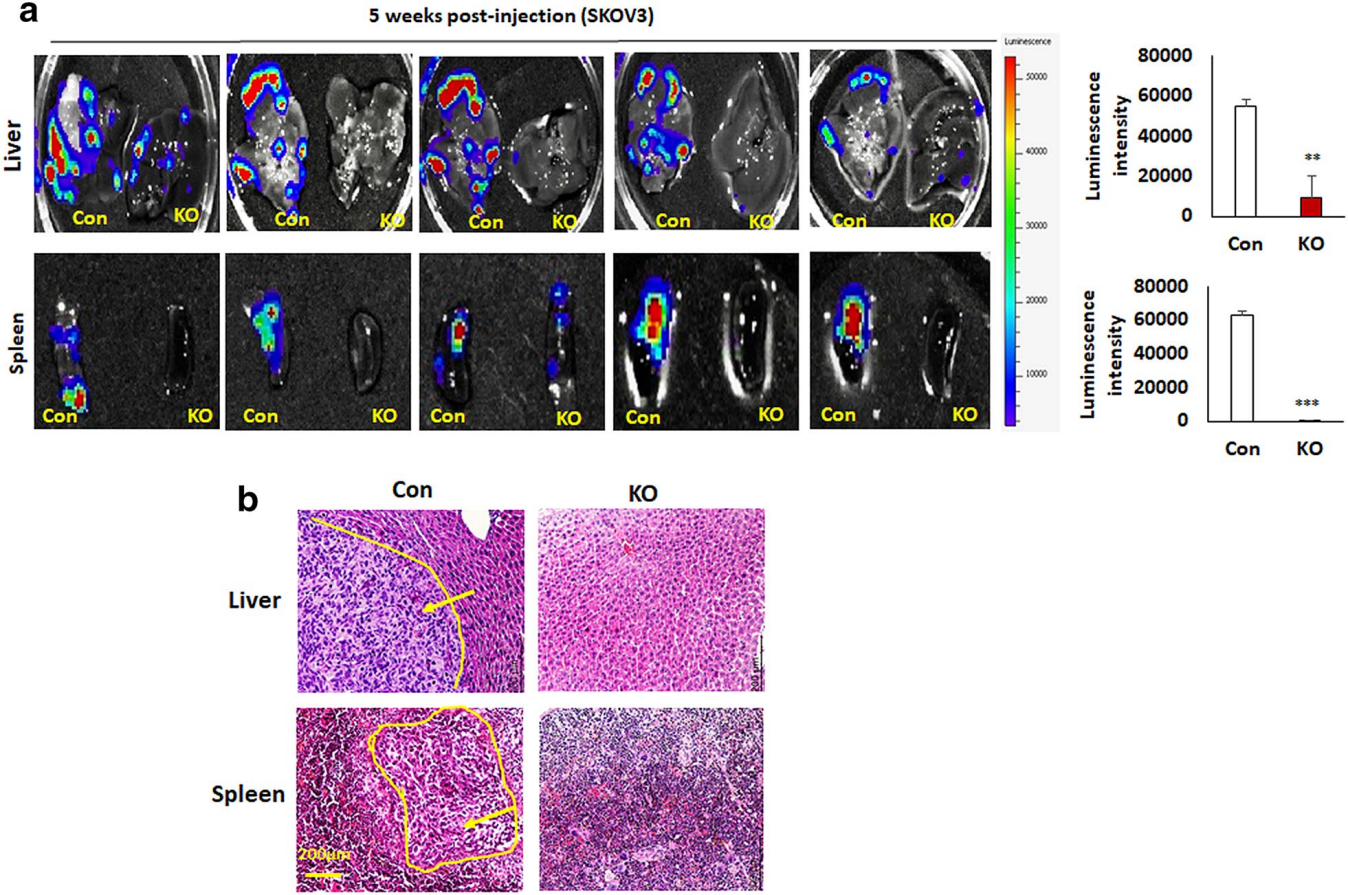
Knockout of EIF5A2 using lentiviral CRISPR/Cas9 nickase vector suppressed ovarian tumor metastasis in an orthotopic ovarian mouse model. **a** Metastatic tumors in liver and spleen of mice xenografted with EIF5A2 KO and control (Con) cells. **b** Sections of metastatic tumors in liver and spleen were stained with H&E. Arrow indicates tumor area

To determine whether EIF5A2 contributes to primary ovarian tumor growth and metastasis in vivo, we injected 5 × 10^5^ ovarian cancer SKOV3 KO and control cells intrabursally into two-month-old immunocompromised NSG female mice. The primary ovarian tumors were significantly reduced in mice injected with EIF5A2 KO cells than control mice as indicated by tumor weight (Fig. 7a). We then examined EIF5A2, EMT markers and pSMAD2 expression in primary ovarian tumors by WB. We found that EIF5A2, and the mesenchymal markers β-catenin, snail2, and vimentin and pSMAD2 were downregulated, whereas the epithelial markers cytokeratin-7 and E-cadherin were upregulated in ovarian tumors of mice implanted with EIF5A2 KO SKOV3 compared to control cells (Fig. 7b). Ovarian tumors were also evaluated by H&E staining (Fig. 7c). Ovarian tumor sections were immunostained with EIF5A2, vimentin, and cytokeratin-7 antibodies. EIF5A2 and vimentin showed weak staining, whereas cytokerain-7 staining was strong in tumors from mice xenografted with KO cells compared with control cells (Additional file 1: Fig. S2a–c). Furthermore, we found metastatic tumors in multiple peritoneal organs including the liver and spleen of mice injected with control cells, but fewer metastasis was found in mice implanted with EIF5A2 KO cells as shown by bioluminescence and verified using H&E staining (Fig. 8a, b). Our results indicated that loss of EIF5A2 suppressed primary ovarian tumor growth and tumor metastasis by inhibiting EMT and attenuating the TGFβ pathway in orthotopic ovarian cancer mouse models.

## Discussion

Lack of effective predictive biomarkers is a major issue in early diagnosis of ovarian cancer patients. We report that EIF5A2 is a potential predictive biomarker for early diagnosis of OC and prognosis following chemotherapy. In particular, EIF5A2 is correlated with the poor survival of ovarian cancer patients. For the first time, we demonstrated in this study that EIF5A2 contributes to ovarian tumor metastasis by promoting EMT via activation of the TGFβ pathway. EIF5A2 was shown to associate with metastasis, developmental stages, histological types and poor patient survival in gall bladder cancer [34], oral squamous cell carcinoma [35], prostate cancer [36], cervical cancer [37], and hepatocellular carcinoma [38]. In the present study, we analyzed the expression of EIF5A2 in high grade serous ovarian carcinoma from several databases, and found EIF5A2 was associated with tumor metastasis and poor patient survival. However, it remains to be further analyzed whether there is correlation between EIF5A2 expression level and the different stages and/or grades in other types of OC including clear cell, endometrioid, and mucinous carcinomas. Our studies indicate that EIF5A2 is a potential biomarker for diagnosis and prognosis and also an attractive drug target due to its low expression in normal tissues and high expression in ovarian tumors.

Using gain- and loss-of-function approaches through lentiviral vector-based gene editing and overexpression, we showed for the first time that EIF5A2 promotes EMT in ovarian cancer cells, suggesting that it may contribute to ovarian cell invasion and metastasis. In support of this hypothesis, we found that KO of EIF5A2 not only inhibited primary ovarian tumor growth and clonogenicity but also ovarian tumor metastasis in orthotopic ovarian cancer mouse models (Figs. 7 and 8). Our finding is consistent with previous studies reporting that EIF5A2 promoted EMT and contributed to cell invasion, chemoresistance, and metastasis in several cancer types including HCC [39], colorectal cancer [15], bladder cancer [40], and OSCC [35]. Therefore, targeting EIF5A2 may inhibit tumor metastasis and overcome chemoresistance by reversing EMT in ovarian cancer cells. EIF5A2 is the only known hypusinated protein and matures through processing by a hypusination pathway and via a rate -limiting first step by DHS required for EIF5A2 maturation. A small molecular inhibitor of DHS, GC-7 has been used to disrupt the hypusination pathway. GC-7 has been shown to inhibit EMT in hepatocellular carcinoma [41], bladder cancer [40] and breast cancer [17]. We propose that inhibition of EIF5A2 maturation using GC-7 may suppress tumor metastasis by reversing EMT [17, 42]. Therefore, targeting EIF5A2 hypusination using DHS inhibitors might be a new approach for therapy of OC.

Although we showed that EIF5A2 promoted EMT in ovarian cancer cells, the molecular mechanisms by which EIF5A2 regulates EMT remains unclear. We previously showed that TGFβ promotes EMT in ovarian cancer [27]. Interestingly, we found that TGFβ induced EIF5A2 expression, whereas inhibition of TGFβR1/2 using SB431542 or knockdown of TGFβR2 suppressed EIF5A2 expression. KO of EIF5A2 attenuated TGFβ pathway, while overexpressing EIF5A2 activated TGFβ pathways. Our studies indicated a positive feedback loop between EIF5A2 and the TGFβ pathway in ovarian cancer cells. Previous studies also showed that EIF5A2 is involved in TGFβ pathway [40, 43]. However, EIF5A2 is negatively correlated with TGFβ signaling in anaplastic thyroid carcinoma [43]. In contrast, EIF5A2 was correlated positively with TGFβ signaling in bladder cancer by stabilizing STAT3 binding to the TGFR1 promoter [40]. EIF5A2 was also post-transcriptionally upregulated by hnRNPE1 through binding 3′ untranslated region in a TGFβ-dependent manner in NMuMG cells [44]. Our studies indicate a positive feed-back loop between EIF5A2 and TGFβ signaling pathway, which may contribute to OC invasion and metastasis by promoting EMT. However, it is still unclear how EIF5A2 interacts with TGFβ pathway. Based on the results of our luciferase reporter gene assay, it appears that SMAD2/3/4 may bind the promoter of EIF5A2 and activate EIF5A2 expression indicated by TGFβ-induced luciferase reporter expression in EIF5A2 expressing cells, but not the EIF5A2 KO cells (Fig. 6).

## Conclusion

Our study demonstrated that EIF5A2 is highly expressed in ovarian HGSC and associated with patient poor survival. EIF5A2 promotes primary ovary tumor growth and metastasis by promoting EMT and activating the TGFβ pathway.

## Supplementary Information

**Additional file 1: Fig. S1.** EIF5A2 expression in ovarian cancer cell line and tissues.**a**. Endogenous EIF5A2 expression in OVCAR3, SKOV3 and OVCAR8 cells. ** b**. OC sections immunofluorescent stained with EIF5A2 (green) and PCNA (Red) antibodies and cell nuclei were counterstained with DAPI (blue). ** c** EIF5A2 expression is significantly higher in the high-risk group than that in the low risk group in 415 ovarian carcinoma samples in the SurvExpress database. (***p<0.001).** Fig. S2.** EIF5A2 and EMT markers were stained in sections of ovarian tumor of EIF5A2 KO and control mice. **a–c ** OC sections were stained by EIF5A2, Vimentin and cytokeratin-7 antibodies (green) and for cell proliferation was stained with PCNA antibody(red). Cell nuclei were counterstained with DAPI. Sections were also stained with H&E.

## Data Availability

The data and materials generated during the current study are available from the corresponding author.

## Abbreviations

EMT: Epithelial to mesenchymal transition
OC: Ovarian cancer
EIF5A2: Eukaryotic initiation factor 5A
DHPS: Deoxyhypusine synthase
DOHH: Deoxyhypusine hydroxylase
KO: Knockout
KD: Knockdown
CRISPR: Clustered regularly interspaced short palindromic repeats
CPTAC: Clinical Proteomic Tumor Analysis Consortium

## References

[1] Siegel RL, Miller KD, Jemal A (2019). Cancer statistics, 2019. CA Cancer J Clin.

[2] Terraneo N, Jacob F, Dubrovska A, Grunberg J (2020). Novel therapeutic strategies for ovarian cancer stem cells. Front Oncol.

[3] Scalici JM, Arapovic S, Saks EJ, Atkins KA, Petroni G, Duska LR, Slack-Davis JK (2017). Mesothelium expression of vascular cell adhesion molecule-1 (VCAM-1) is associated with an unfavorable prognosis in epithelial ovarian cancer (EOC). Cancer.

[4] Ahmed N, Stenvers KL (2013). Getting to know ovarian cancer ascites: opportunities for targeted therapy-based translational research. Front Oncol.

[5] Lengyel E (2010). Ovarian cancer development and metastasis. Am J Pathol.

[6] Park MH, Nishimura K, Zanelli CF, Valentini SR (2010). Functional significance of eIF5A and its hypusine modification in eukaryotes. Amino Acids.

[7] Fujimura K, Choi S, Wyse M, Strnadel J, Wright T, Klemke R (2015). Eukaryotic translation initiation factor 5A (EIF5A) regulates pancreatic cancer metastasis by modulating RhoA and Rho-associated kinase (ROCK) protein expression levels. J Biol Chem.

[8] Mathews MB, Hershey JW (2015). The translation factor eIF5A and human cancer. Biochim Biophys Acta.

[9] Wang FW, Guan XY, Xie D (2013). Roles of eukaryotic initiation factor 5A2 in human cancer. Int J Biol Sci.

[10] Clement PM, Henderson CA, Jenkins ZA, Smit-McBride Z, Wolff EC, Hershey JW, Park MH, Johansson HE (2003). Identification and characterization of eukaryotic initiation factor 5A–2. Eur J Biochem.

[11] Jenkins ZA, Haag PG, Johansson HE (2001). Human eIF5A2 on chromosome 3q25-q27 is a phylogenetically conserved vertebrate variant of eukaryotic translation initiation factor 5A with tissue-specific expression. Genomics.

[12] Pallmann N, Braig M, Sievert H, Preukschas M, Hermans-Borgmeyer I, Schweizer M, Nagel CH, Neumann M, Wild P, Haralambieva E (2015). Biological relevance and therapeutic potential of the hypusine modification system. J Biol Chem.

[13] He LR, Zhao HY, Li BK, Liu YH, Liu MZ, Guan XY, Bian XW, Zeng YX, Xie D (2011). Overexpression of eIF5A-2 is an adverse prognostic marker of survival in stage I non-small cell lung cancer patients. Int J Cancer.

[14] Shek FH, Fatima S, Lee NP (2012). Implications of the use of eukaryotic translation initiation factor 5A (eIF5A) for prognosis and treatment of hepatocellular carcinoma. Int J Hepatol.

[15] Zhu W, Cai MY, Tong ZT, Dong SS, Mai SJ, Liao YJ, Bian XW, Lin MC, Kung HF, Zeng YX (2012). Overexpression of EIF5A2 promotes colorectal carcinoma cell aggressiveness by upregulating MTA1 through C-myc to induce epithelial-mesenchymaltransition. Gut.

[16] Sun J, Xu Z, Lv H, Wang Y, Wang L, Ni Y, Wang X, Hu C, Chen S, Teng F (2018). eIF5A2 regulates the resistance of gastric cancer cells to cisplatin via induction of EMT. Am J Transl Res.

[17] Liu Y, Liu R, Fu P, Du F, Hong Y, Yao M, Zhang X, Zheng S (2015). N1-Guanyl-1,7-diaminoheptane sensitizes estrogen receptor negative breast cancer cells to doxorubicin by preventing epithelial-mesenchymal transition through inhibition of eukaryotic translation initiation factor 5A2 activation. Cell Physiol Biochem.

[18] Drasin DJ, Robin TP, Ford HL (2011). Breast cancer epithelial-to-mesenchymal transition: examining the functional consequences of plasticity. Breast Cancer Res.

[19] DiMeo TA, Anderson K, Phadke P, Fan C, Perou CM, Naber S, Kuperwasser C (2009). A novel lung metastasis signature links Wnt signaling with cancer cell self-renewal and epithelial-mesenchymal transition in basal-like breast cancer. Cancer Res.

[20] Micalizzi DS, Farabaugh SM, Ford HL (2010). Epithelial-mesenchymal transition in cancer: parallels between normal development and tumor progression. J Mammary Gland Biol Neoplasia.

[21] Ahmed N, Thompson EW, Quinn MA (2007). Epithelial-mesenchymal interconversions in normal ovarian surface epithelium and ovarian carcinomas: an exception to the norm. J Cell Physiol.

[22] Bozhkova DM, Poryazova-Markova EG (2019). The epithelial-mesenchymal transition, E-cadherin and tumor progression in ovarian serous tumors. Folia Med (Plovdiv).

[23] Bhuyan G, Arora R, Ahluwalia C, Sharma P (2019). Epithelial-mesenchymal transition in serous and mucinous epithelial tumors of the ovary. J Cancer Res Ther.

[24] Antony J, Thiery JP, Huang RY (2019). Epithelial-to-mesenchymal transition: lessons from development, insights into cancer and the potential of EMT-subtype based therapeutic intervention. Phys Biol.

[25] Solheim O, Forsund M, Trope CG, Kraggerud SM, Nesland JM, Davidson B (2017). Epithelial-mesenchymal transition markers in malignant ovarian germ cell tumors. APMIS.

[26] Rafehi S, Ramos Valdes Y, Bertrand M, McGee J, Prefontaine M, Sugimoto A, DiMattia GE, Shepherd TG (2016). TGFbeta signaling regulates epithelial-mesenchymal plasticity in ovarian cancer ascites-derived spheroids. Endocr Relat Cancer.

[27] Chen Z, Wang Y, Liu W, Zhao G, Lee S, Balogh A, Zou Y, Guo Y, Zhang Z, Gu W (2014). Doxycycline inducible Kruppel-like factor 4 lentiviral vector mediates mesenchymal to epithelial transition in ovarian cancer cells. PLoS ONE.

[28] Zhao G, Wang Q, Wu Z, Tian X, Yan H, Wang B, Dong P, Watari H, Pfeffer LM, Guo Y (2019). Ovarian primary and metastatic tumors suppressed by survivin knockout or a novel survivin inhibitor. Mol Cancer Ther.

[29] Yue J, Sheng Y, Ren A, Penmatsa S (2010). A miR-21 hairpin structure-based gene knockdown vector. Biochem Biophys Res Commun.

[30] Zhao G, Wang Q, Gu Q, Qiang W, Wei JJ, Dong P, Watari H, Li W, Yue J (2017). Lentiviral CRISPR/Cas9 nickase vector mediated BIRC5 editing inhibits epithelial to mesenchymal transition in ovarian cancer cells. Oncotarget.

[31] Kandoth C, McLellan MD, Vandin F, Ye K, Niu B, Lu C, Xie M, Zhang Q, McMichael JF, Wyczalkowski MA (2013). Mutational landscape and significance across 12 major cancer types. Nature.

[32] Gyorffy B, Lanczky A, Szallasi Z (2012). Implementing an online tool for genome-wide validation of survival-associated biomarkers in ovarian-cancer using microarray data from 1287 patients. Endocr Relat Cancer.

[33] Aguirre-Gamboa R, Gomez-Rueda H, Martinez-Ledesma E, Martinez-Torteya A, Chacolla-Huaringa R, Rodriguez-Barrientos A, Tamez-Pena JG, Trevino V (2013). SurvExpress: an online biomarker validation tool and database for cancer gene expression data using survival analysis. PLoS ONE.

[34] Zheng X, Gao L, Wang BT, Shen P, Yuan XF, Zhang LQ, Yang L, Zhang DP, Zhang Q, Wang XM (2020). Overexpression of EIF5A2 is associated with poor survival and aggressive tumor biology in gallbladder cancer. Histol Histopathol.

[35] Lin YM, Chen ML, Chen CL, Yeh CM, Sung WW: Overexpression of EIF5A2 Predicts Poor Prognosis in Patients with Oral Squamous Cell Carcinoma. *Diagnostics (Basel)* 2020, 10(7). 10.3390/diagnostics10070436 PMC7400414 32605067

[36] Lu J, Zhao HW, Chen Y, Wei JH, Chen ZH, Feng ZH, Huang Y, Chen W, Luo JH, Fang Y (2019). Eukaryotic translation initiation factor 5A2 is highly expressed in prostate cancer and predicts poor prognosis. Exp Ther Med.

[37] Yang SS, Gao Y, Wang DY, Xia BR, Liu YD, Qin Y, Ning XM, Li GY, Hao LX, Xiao M (2016). Overexpression of eukaryotic initiation factor 5A2 (EIF5A2) is associated with cancer progression and poor prognosis in patients with early-stage cervical cancer. Histopathology.

[38] Lee NP, Tsang FH, Shek FH, Mao M, Dai H, Zhang C, Dong S, Guan XY, Poon RT, Luk JM (2010). Prognostic significance and therapeutic potential of eukaryotic translation initiation factor 5A (eIF5A) in hepatocellular carcinoma. Int J Cancer.

[39] Tang DJ, Dong SS, Ma NF, Xie D, Chen L, Fu L, Lau SH, Li Y, Li Y, Guan XY (2010). Overexpression of eukaryotic initiation factor 5A2 enhances cell motility and promotes tumor metastasis in hepatocellular carcinoma. Hepatology.

[40] Wei JH, Cao JZ, Zhang D, Liao B, Zhong WM, Lu J, Zhao HW, Zhang JX, Tong ZT, Fan S (2014). EIF5A2 predicts outcome in localised invasive bladder cancer and promotes bladder cancer cell aggressiveness in vitro and in vivo. Br J Cancer.

[41] Lou B, Fan J, Wang K, Chen W, Zhou X, Zhang J, Lin S, Lv F, Chen Y (2013). N1-guanyl-1,7-diaminoheptane (GC7) enhances the therapeutic efficacy of doxorubicin by inhibiting activation of eukaryotic translation initiation factor 5A2 (eIF5A2) and preventing the epithelial-mesenchymal transition in hepatocellular carcinoma cells. Exp Cell Res.

[42] Zhou QY, Tu CY, Shao CX, Wang WK, Zhu JD, Cai Y, Mao JY, Chen W (2017). GC7 blocks epithelial-mesenchymal transition and reverses hypoxia-induced chemotherapy resistance in hepatocellular carcinoma cells. Am J Transl Res.

[43] Huang Y, Zhu Q, Lu L, Sun S, Hao F, Zhang J, Liu Z, Miao Y, Jiao X, Chen D: EIF5A2 is highly expressed in anaplastic thyroid carcinoma and is associated with tumor growth by modulating TGF-beta signals. Oncol Res 2020. 10.3727/096504020X15834065061807 PMC7851513 32138807

[44] Hussey GS, Link LA, Brown AS, Howley BV, Chaudhury A, Howe PH (2012). Establishment of a TGFbeta-induced post-transcriptional EMT gene signature. PLoS ONE.

