# Supplementary material for: EIF5A2 controls ovarian tumor growth and metastasis by promoting epithelial to mesenchymal transition via the TGFβ pathway
Source: Cell Biosci. 2021 Apr 7;11:70. doi: 10.1186/s13578-021-00578-5 (PMC8025533; doi:10.1186/s13578-021-00578-5)
Supplement: Supplementary file 1 — Additional file 1: Fig. S1. EIF5A2 expression in ovarian cancer cell line and tissues.a. Endogenous EIF5A2 expression in OVCAR3, SKOV3 and OVCAR8 cells. b. OC sections immunofluorescent stained with EIF5A2 (green) and PCNA (Red) antibodies and cell nuclei were counterstained with DAPI (blue). c EIF5A2 expression is significantly higher in the high-risk group than that in the low risk group in 415 ovarian carcinoma samples in the SurvExpress database. (***p<0.001). Fig. S2. EIF5A2 and EMT markers were stained in sections of ovarian tumor of EIF5A2 KO and control mice. a–c OC sections were stained by EIF5A2, Vimentin and cytokeratin-7 antibodies (green) and for cell proliferation was stained with PCNA antibody(red). Cell nuclei were counterstained with DAPI. Sections were also stained with H&E. [file 13578_2021_578_MOESM1_ESM.docx]

**EIF5A2 controls ovarian tumor growth and metastasis by promoting epithelial to mesenchymal transition via the TGFβ pathway**

Guannan Zhao^1,2^, Wenjing Zhang^3^, Peixin Dong^4^, Hidemichi Watari^4^, *Yuqi Guo^5^, Lawrence M. Pfeffer^1,2^, Gabor Tigyi^6^, Junming Yue^1,2*^

^1^Department of Pathology and Laboratory Medicine, ^2^Center for Cancer Research, College of Medicine, the University of Tennessee Health Science Center, Memphis, TN, 38163, USA; [gzhao4@uthsc.edu](mailto:gzhao4@uthsc.edu) (G.Z.); [jyue@uthsc.edu](mailto:jyue@uthsc.edu) (J.Y.); lpfeffer@uthsc.edu (L.P.)

^3^Department of Genetics, Genomics & Informatics, College of Medicine, the University of Tennessee Health Science Center, Memphis, TN, 38163, USA; [wzhang67@uthsc.edu](mailto:wzhang67@uthsc.edu) (W.Z.)

^4^Department of Obstetrics and Gynecology, Hokkaido University Graduate School of Medicine, Sapporo, 060-8638, Japan; [dpx1cn@gmail.com](mailto:dpx1cn@gmail.com) (P.D.); [watarih@med.hokudai.ac.jp](mailto:watarih@med.hokudai.ac.jp) (H.W.)

^5^People's Hospital of Zhengzhou University, Zhengzhou, Henan, China. [yuqi-guo@163.com](mailto:yuqi-guo@163.com) (Y. G.)

^6^Department of Physiology, College of Medicine, the University of Tennessee Health Science Center, Memphis, TN, 38163, USA; [gtigyi@uthsc.edu](mailto:gtigyi@uthsc.edu) (G.T.)

* Correspondences: [jyue@uthsc.edu](mailto:jyue@uthsc.edu) (Y.J.) or [yuqi-guo@163.com](mailto:yuqi-guo@163.com) (Y.Q.)


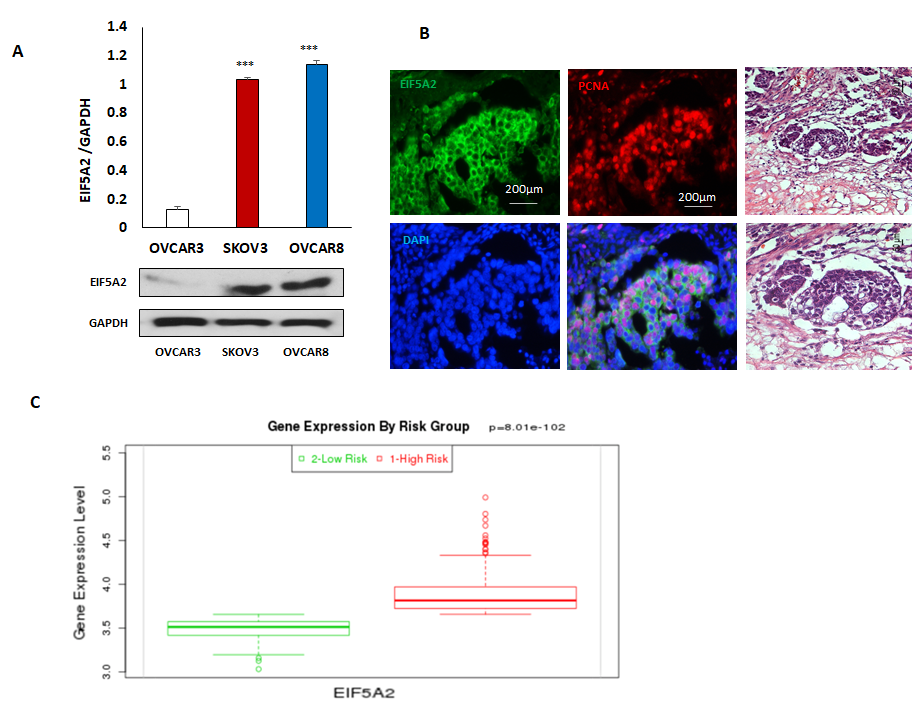


**Fig. S1. EIF5A2 expression in ovarian cancer cell line and tissues.** **A.** Endogenous EIF5A2 expression in OVCAR3, SKOV3 and OVCAR8 cells**. B.** OC sections immunofluorescent stained with EIF5A2 (green) and PCNA (Red) antibodies and cell nuclei were counterstained with DAPI (blue). **C.** EIF5A2 expression is significantly higher in the high-risk group than that in the low risk group in 415 ovarian carcinoma samples in the SurvExpress database**.** (***p<0.001).


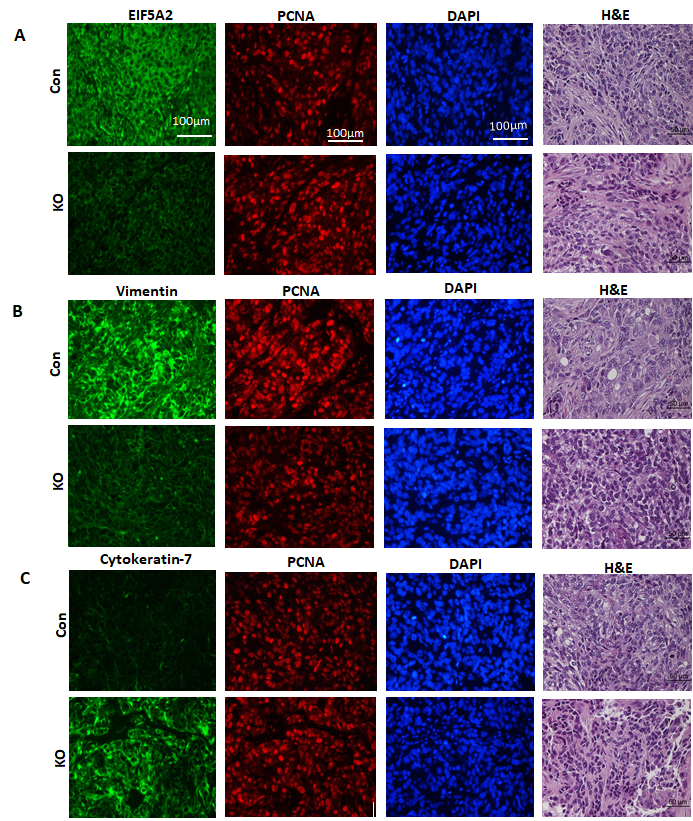


**Fig. S2. EIF5A2 and EMT markers were stained in sections of ovarian tumor of EIF5A2 KO and control mice. A, B, C:** OC sections were stained by EIF5A2, Vimentin and cytokeratin-7 antibodies (green) and for cell proliferation was stained with PCNA antibody(red). Cell nuclei were counterstained with DAPI. Sections were also stained with H&E.
